# Pharmacotherapy problems in cardiology patients 30 days post discharge from a tertiary hospital in Brazil: a randomized controlled trial

**DOI:** 10.6061/clinics/2019/e1091

**Published:** 2019-11-19

**Authors:** Aline F. Bonetti, Bruna Q. Bagatim, Wallace Entringer Bottacin, Antonio M. Mendes, Inajara Rotta, Renata C. Reis, Maria Luiza D. Fávero, Fernando Fernandez-Llimos, Roberto Pontarolo

**Affiliations:** I Programa de Pos-Graduacao em Ciencias Farmaceuticas, Universidade Federal do Parana, Curitiba, PR, BR; II Department of Social Pharmacy, Institute for Medicines Research (iMed. ULisboa), Faculty of Pharmacy, University of Lisbon, Lisbon, Portugal

**Keywords:** Patient Discharge, Counseling, Pharmacists

## Abstract

**OBJECTIVES:**

This is a randomized controlled trial that aims to evaluate the impact of pharmacist-led discharge counseling on reducing pharmacotherapy problems in the 30-day postdischarge period of cardiology patients from a tertiary hospital in Brazil.

**METHODS:**

At discharge, two cardiovascular pharmacy residents performed a medication counseling session with the intervention group, and the follow-up was performed by telephone (3 and 15 days after discharge). The number of pharmacotherapy problems was evaluated during a pharmacist-led ambulatory consultation 30 days after discharge.

**RESULTS:**

A total of 66 and 67 patients were randomized to the intervention and control groups, respectively, but only 51 patients were analyzed in each group, all with similar baseline characteristics. The intervention group had significantly fewer pharmacotherapy problems compared to the control (*p*<0.001), and 100% of the patients had at least one problem. We observed five problems significantly more frequently in the control group: “incorrect time of taking” (*p*=0.003), “use higher dose of medication” (*p*=0.007), “use lower dose of medication” (*p*=0.014), “restart discontinued medication” (*p*=0.011), and “underdosing prescription” (*p*=0.009). Simvastatin, enalapril, carvedilol, and atorvastatin were the medications more associated with pharmacotherapy problems.

**CONCLUSIONS:**

We concluded that pharmacist-led discharge counseling should be an indispensable service, as patients exhibited less pharmacotherapy problems in the 30-day postdischarge period, especially related to drug administration and adherence.

## INTRODUCTION

Medication errors related to hospital discharge constitute an important risk factor for rehospitalization (1). There are several elements that contribute to increased readmission rates, including the time to primary care follow-up, adverse drug events (ADEs) (2-4), medication nonadherence (5,6), medication discrepancies (7,8), insufficient discharge planning (9), and lack of continuity care (10). A retrospective study demonstrated that 100% of the patients had at least one ADE following hospital discharge, including minor events, such as the need for laboratory monitoring or medication prescribed without a listed indication, and several potentially life-threatening events, including repeated occurrences of hypoglycemia, altered mental status, and critical laboratory values.

Medication discrepancies, defined as differences between what patients think they should be taking and regimens ordered by physicians (4), may occur when patients are admitted or discharged from a hospital (11). Following discharge, inaccuracies in the discharge medication list and lack of communication between patients and health professionals may contribute to this problem (10).

Previous studies have shown that elderly polypharmacy patients frequently have drug-related problems, which may cause hospital admissions (12-14). Discrepancies may include inappropriate medication omission, wrong dose, incorrect duration, medication without a known indication, and inappropriate drug continuation (8).

Coleman et al. demonstrated that patients with medication discrepancies had a 30-day hospital readmission rate of 14.3%, compared with 6.1% for patients without discrepancies (*p*=0.04). Patients who presented any discrepancy had used significantly more medications than those who did not (9.0 *vs*. 7.1, *p*=0.001); most were 65 years old and older and presented cardiology diseases, especially congestive heart failure. The five medication classes most involved with these problems were anticoagulants (13%), diuretics (10%), angiotensin-converting enzyme inhibitors (10%), lipid-lowering agents (10%), and proton pump inhibitors (7%) (7).

Medication review, discharge medication counseling, and telephone follow-up performed by pharmacists have been associated with a significantly lower rate of preventable ADEs in the 30-day postdischarge period that were related to discrepancies, inappropriate prescribing, lack of medication access, nonadherence, and inadequate drug monitoring (4). Identifying medication discrepancies during hospital admission (11) and during medication reconciliation and discharge is an important role of the clinical pharmacist. Other studies have suggested that medication counseling prior to discharge also improves adherence (5,15), decreases readmission rates (1,16), and mortality (5) and consequently promotes cost savings (16-18).

Given that discharge medication counseling can reduce discrepancies, ADEs, and other drug-related problems and that most of the studies were conducted in high income countries and evaluated the endpoints by telephone, the aim of this controlled trial was to assess the impact of a pharmacist-led medication counseling session on pharmacotherapy problems in the 30-day postdischarge period.

## METHODS

This study presents a randomized controlled trial conducted in a tertiary hospital of Curitiba, Brazil, from February to December 2015. Patients aged 18 years and older were eligible if they were admitted to a specialized cardiology ward due to stable angina, acute coronary syndrome, congestive heart failure, valvular disease, arrhythmias, or hypertension. The exclusion criteria were patients with cognitive impairment and without a caregiver, patients in palliative care, patients transferred to other clinical specialties or institutions, and patients not discharged from the hospital during the study.

Participants were assigned following randomization procedures to one of two groups in a 1:1 ratio. A random number list was generated on Microsoft Office Excel 2010^®^ by a third person who was not involved in the study to guarantee the blindness of the concealment. When the patient was admitted to the hospital according to the eligibility criteria, he/she was immediately allocated to this random list according to the sequence previously generated. The sample size was calculated considering 5.0% type I and 20% type II error (80% power) rates based on hospital readmission rates reported in the literature, resulting in 53 patients in each group.

For the intervention group, the pharmacist reviewed the discharge prescription and discussed significant findings with the medical team to ensure medication use followed evidence from guidelines. As necessary, a change in prescription was made at this time. After that, the patients, or their caregivers, received individual counseling sessions by the pharmacist, which included explanations about the indications, benefits, therapeutic targets, doses, dosing schedule, routes, storage, length of therapy, refill pharmacy, and possible ADEs of each prescribed drug. A leaflet containing the information from the verbal counseling was provided and delivered by pharmacists to optimize the medication adherence. Afterwards, patients were contacted by telephone by the same pharmacist at 3 and 15 days post discharge to reinforce the previous counseling session. All pharmacist interventions were performed according to a novel tool entitled Descriptive Elements of Pharmacist Interventions Characterization Tool (DEPICT) (19). The control group received usual care by the pharmacists and other healthcare providers. Both groups were scheduled for an appointment 30 days post discharge at the Pharmaceutical Care Ambulatory of the same hospital to collect data regarding the outcomes. For more detailed methods, see the original article (20).

The endpoints were the number of pharmacotherapy problems and the medications related to the problems. We defined “pharmacotherapy problems” as any problem related to pharmacotherapy, including problems with a prescription, drug administration and adherence, discrepancy between healthcare settings, need for monitoring, ineffective treatment, and ADEs, which could compromise the therapeutic results. At the Pharmaceutical Care Ambulatory, after identifying the problem, the pharmacist performed the intervention for both groups as necessary, including suggestions of pharmacotherapy changes (by writing a letter to the prescriber), pharmaceutical counseling, laboratory test requests, and recommendations for self-monitoring. The pharmaceutical consultation returns were scheduled as required, and the therapeutic follow-up was conducted for all patients to solve the previously discovered pharmacotherapy problems.

Statistical analyses were conducted using Statistica version 10.0. The Kolmogorov-Smirnov test was performed to assess the normality of the distribution of the investigated parameters. Student’s *t-*tests and Mann-Whitney tests were performed for continuous data as appropriate, while categorical variables were assessed with the chi-square test. For qualitative analysis of the pharmacotherapy problems in both groups, the chi-square test was also performed, considering the presence or absence of the problems. We performed *intention-to-treat* (ITT) analyses and considered *p-*values less than 0.05 to be statistically significant.

This trial was in accordance with the ethical standards of the institution’s committee (registration number: 40431015.8.0000.0096).

## RESULTS

**Figure 1:**
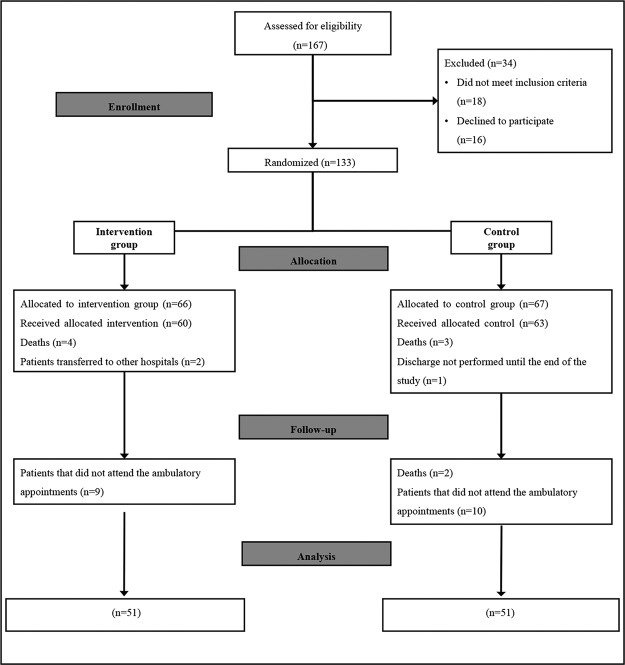
Flow diagram of the intervention and control groups of the randomized trial.

Among 167 eligible patients recruited from February 2015 to November 2015, 133 patients volunteered to participate, and 34 were excluded. A total of 66 and 67 patients were allocated to the intervention and control groups, respectively, and follow-up occurred until December 2015. At the end of the study, 51 patients remained in each group (Figure 1).

There were no statistically significant differences in the patient baseline characteristics, except for hypertension and dyslipidemia, which were clinical conditions more prevalent in the intervention group than in the control group (Table 1). The mean age of the participants was 67 and 66 years in the control and intervention groups, respectively. The majority of the patients were male, on polypharmacy, and the most common comorbidities were hypertension, diabetes, coronary artery disease, and dyslipidemia (Table 2).

A total of 356 pharmacotherapy problems were identified, with 276 and 80 in the control and intervention groups, respectively. All patients had at least one problem, although the individuals in the control group had significantly more than the individuals in the intervention group [4 (±4.2) *vs*. 1 (±1.5), *p*<0.001]. The majority of identified problems were related to drug administration and adherence, especially in the control group (n=148 *vs*. n=19), followed by prescription errors (n=72 *vs*. n=30).

The most frequent problems in the control group, which were statistically significant compared with the intervention group, were “incorrect time of taking”, “use lower dose of medication”, “use higher dose of medication”, “restart discontinued medication”, and “underdosing prescription” (Table 3). The drugs most commonly involved with the identified problems were simvastatin (6.74%), enalapril (6.74%), carvedilol (5.99%), atorvastatin (5.62%), aspirin (5.24%), and psychiatric agents (5.24%).

Other frequent problems were related to monitoring (especially self-monitoring and the need for laboratory tests) and “clinical health condition without treatment”, but the differences between the groups were not significant (Table 3).

Additionally, our previous publication about the same study showed that the intervention group had a significantly lower readmission rate related to heart disease than the control group (0% *vs*. 11.3%, *p*=0.027) (20). Therefore, the incidence of pharmacotherapy problems in the control group, especially those problems with higher clinical significance, could contribute to hospital readmission.

## DISCUSSION

According to Cipolle et al., a medication-related problem [or drug-related problem (DRP)] occurs when a patient experiences or is likely to experience a disease or a symptom having an actual or suspected relationship with pharmacotherapy (21). Moreover, Varkey et al. defined medication discrepancy as a difference in drug name, dose, frequency, and mode of administration between the medical record and the patient-generated report (22). In this study, “pharmacotherapy problems” included all problems related to medications, which included the concept of medication-related problems and medication discrepancy.

According to Bech et al., DRPs with higher clinical relevance require medical consultation or hospital admission, while DRPs with lower clinical relevance can be solved by other health professionals and do not require medical attention, e.g., by pharmacist-provided patient counseling on the correct use of the medicine (23). In this study, most pharmacotherapy problems with higher clinical relevance were those related to prescriptions errors, which required medical consultation to solve them. As mentioned above, the control group had more clinically relevant pharmacotherapy problems than the intervention group (n=72 *vs*. n=30).

The results of this study demonstrate the contribution of the pharmacist in reducing pharmacotherapy problems 30 days after hospital discharge, especially problems related to incorrect time of taking the drug, using lower and higher doses, restarting a discontinued medication, and underdosing prescription. “Clinical condition without treatment” and “need for monitoring”, especially self-monitoring, were frequent problems identified in the 30-day postdischarge period, indicating the importance of follow-up of cardiology patients to identify pharmacotherapy problems. Moreover, 100% of patients had at least one pharmacotherapy problem since most of them were elderly polypharmacy patients, representing a population susceptible to DRPs (12), and consequently to hospital readmission (7,8).

According to Farley et al., clinically important medication discrepancies in the primary care physician record were different between patients who received a clinical pharmacist’s intervention during hospital admission and discharge. However, this effect was sustained at 30 days post discharge (*p*=0.013) but not at 90 days (11). Our study confirms the need for involving pharmacists at hospital discharge, as patients who received medication counseling had significantly less pharmacotherapy problems than patients with standard care in the 30 days after discharge, especially with respect to drug administration and adherence.

A descriptive analysis from a larger randomized trial demonstrated that the mean number of DRPs was similar to that in the control group in the present study, 3.9 (±3.2), and the most prevalent was “patient requires drug therapy”, which was frequently involved with respiratory, cardiovascular, and musculoskeletal systems (13). Another randomized controlled trial, performed only with patients with heart failure, revealed that restarting discontinued medication and using higher doses of medication were discrepancies more common in the control group (24), consistent with our study.

An observational study with more than 400 patients with any pathology demonstrated that the more frequent preventable ADEs were nonadherence and underuse (18%), untreated medical problem (15%), and laboratory test needed (13%), and each patient had an ADE mean of 2.9 (±4). More than half of the ADEs could be attributed to three classes of medications: antihypertensives (23%), hypoglycemics (15%), and psychiatric drugs (11%) (3). We also observed that the most prevalent problems were related to medication administration and nonadherence, and the therapeutic classes most commonly associated with problems were lipid-lowering drugs (simvastatin and atorvastatin), antihypertensives (enalapril and carvedilol), an antiplatelet (aspirin), and psychiatric drugs. Another study found that cardiovascular and lipid-lowering agents were some of the drugs most related to ADEs during the first 30 days after discharge (2).

Limitations of this study include a small patient population and a researcher-led intervention randomized trial, which is inherent to the study design. Other limitations include the relatively short postdischarge follow-up and the inability to extend the results to patients with other clinical issues. However, cardiovascular diseases are one of the major causes of hospitalization and mortality worldwide, and patients with these conditions could benefit from the interventions performed in this study. Moreover, even with a small population and a short follow-up, the discharge medication counseling performed by pharmacists showed a significant reduction in pharmacotherapy problems; however, it will be necessary to conduct additional studies with a longer follow-up to determine if the results remain significant.

## CONCLUSIONS

This study demonstrated the need for a pharmacist to perform discharge medication counseling to reduce pharmacotherapy problems in cardiology patients with polypharmacy, especially the problems related to drug administration and adherence, since these problems were significantly less frequent in the intervention group: “incorrect time of taking”, “use higher dose of medication”, “use lower dose of medication”, “restart discontinued medication”, and “underdosing prescription”. The transition of care is a high-risk situation for many patients, and thus discharge counseling and follow-up are necessary to prevent pharmacotherapy problems and clinical outcomes.

## AUTHOR CONTRIBUTIONS

Bonetti AF and Pontarolo R had full access to all of the data in the study and take responsibility for the integrity of the data and the accuracy of the data analysis. Bonetti AF, Bagatim BQ, Mendes AM, Reis RC and Fávero MLD were responsible for the study concept and design. Bonetti AF, Bagatim BQ and Mendes AM were responsible for the acquisition of data. Bonetti AF, Mendes AM, Bottacin WE and Rotta I were responsible for the statistical analysis and interpretation of data. Bonetti AF, Bagatim BQ, Bottacin WE and Mendes AM were responsible for manuscript drafting. Bonetti AF, Bagatim BQ, Bottacin WE, Mendes AM, Rotta I, Reis RC, Fávero MLD, Fernandez-Llimos F and Pontatolo R were responsible for the critical revision of the manuscript for important intellectual content. Fernandez-Llimos F and Pontarolo R were responsible for the study supervision.

## Figures and Tables

**Table 1:** Baseline patient characteristics.

Variables*	Intervention (n = 66)	Control (n = 67)	*p* **
Age	66 (±10)	67 (±12.2)	0.592
Male, n (%)	43 (55)	40 (59)	0.516
Number of comorbidities	4 (±1.6)	4 (±1.9)	0.879
Number of drugs at discharge	8 (±2.4)	8 (±2.5)	0.637
Number of drugs at ambulatory	8 (±3)	9 (±3)	0.250
Days of hospitalization***	10 (±7.9)	11 (±8.2)	0.390
Presence of caregiver, n (%)	19 (28)	19 (28)	0.963

* Mean and standard deviation were reported for continuous data.

** Statistically significant if *p*<0.05.

*** Two outliers were removed from the analysis because they were hospitalized for more than 40 days while waiting for cardiac surgery.

**Table 2:** Prevalence of comorbidities.

Comorbidities*	Intervention (n = 66)	Control (n = 67)	*p* **
Hypertension	59 (89.4)	51 (76.1)	0.043
Diabetes mellitus	29 (43.9)	32 (47.7)	0.658
Coronary artery disease	44 (66.7)	42 (62.7)	0.631
Dyslipidemia	43 (65.1)	28 (41.8)	0.006
Congestive heart failure	23 (34.8)	31 (46.2)	0.180
Arrhythmias	14 (21.2)	9 (13.4)	0.235
Hypothyroidism	11 (16.6)	15 (22.4)	0.405
Smoking	10 (15.1)	11 (16.4)	0.841
Stroke, previous	8 (12.1)	9 (13.4)	0.821
Benign prostatic hyperplasia	5 (7.5)	4 (5.9)	0.387
Valvulopathies	5 (7.6)	3 (4.5)	0.350
Chronic kidney disease	3 (4.5)	6 (8.9)	0.254
Gout	3 (4.5)	0	0.237
Depression	2 (3.0)	1 (1.5)	0.309
Cardiomyopathies	1 (1.5)	2 (2.9)	0.505
Fibromyalgia	1 (1.5)	2 (2.9)	0.505
Others	8 (12.1)	10 (14.9)	0.636

* The mean and % (in parentheses) were reported for each comorbidity.

** *p*<0.05 indicates significance.

**Table 3:** Number of pharmacotherapy problems.

	Pharmacotherapy problem	Intervention*	Control*	*p* **
Related to the prescription	Clinical condition without treatment	10	14	0.389
	Underdosing prescription	1	9	**0.009**
	Needs additional drug therapy	4	7	0.274
	Availability of more cost-effective alternative therapy	4	7	0.274
	Unnecessary drug therapy	3	6	0.254
	Inappropriate drug	2	5	0.226
	Need for preventive drug	0	4	0.131
	Availability of more effective alternative therapy	2	3	0.507
	Overdosing prescription	0	2	0.483
	Availability of safer alternative therapy	0	2	0.483
	Incorrect treatment duration	1	1	0.748
	Frequency or incorrect administration time	0	1	0.994
	Duplicate therapy	1	1	0.748
	Other pharmacotherapy problems	0	2	0.565
Related to medication administration and medication adherence	Incorrect time of drug taking	2	16	**0.003**
	Use higher dose of medication	3	13	**0.007**
	Use lower dose of medication	3	12	**0.014**
	Discontinuation of prescribed medication	5	9	0.207
	Restart discontinued medication	0	8	**0.011**
	Inappropriate self-medication	1	4	0.187
	Administration of the wrong drug	1	3	0.315
	Incorrect administration technique	2	3	0.507
	Patient did not start treatment	1	3	0.315
	Incorrect treatment duration	0	1	0.994
	Abrupt reduction in dose	0	1	0.994
	Abuse of the drug	0	1	0.994
	Other pharmacotherapy problems	1	3	0.315
Discrepancies between healthcare settings	Omission of prescribed drug	2	4	0.331
	Discrepant drug	0	1	0.994
Monitoring	Need for self-monitoring	10	14	0.389
	Need for laboratory test	9	13	0.371
	Need for nonlaboratory test	2	2	0.685
Other	Adverse drug event	6	6	0.978
	Ineffective treatment	3	6	0.254

* *Intention-to-treat analyses*: considering the number of randomized patients.

** *p*<0.05 indicates significance.
